# Convulsions in Children

**Published:** 1889-09

**Authors:** 


### CONVULSIONS IN CHILDREN.

When a child has a convulsion, or what is commonly called "a fit," attention should be given to the urinary secretion at once. If there is suppression of urine, the child should be put into a warm bath, and made to sweat as speedily as possible. In many cases in which children die from a succession of convulsions, the real cause of death is suppression of urine (a fact which is probably not so generally known as it should be), so that the child really dies of poisoning through the retention of the urinary secretion. When a child is subject to attacks of this character, care should be taken to dress it warmly in flannels, so as to keep up a degree of perspiration most of the time, and hot baths should be administered frequently.

